# Seasonal Variation of Water Quality and Phytoplankton Response Patterns in Daya Bay, China

**DOI:** 10.3390/ijerph8072951

**Published:** 2011-07-15

**Authors:** Cui-Ci Sun, You-Shao Wang, Mei-Lin Wu, Jun-De Dong, Yu-Tu Wang, Fu-Lin Sun, Yan-Ying Zhang

**Affiliations:** 1 State Key Laboratory of Tropical Oceanography, South China Sea Institute of Oceanology, Chinese Academy of Sciences, Guangzhou 510301, China; 2 Marine Biology Research Station at Daya Bay, Chinese Academy of Sciences, Shenzhen 518121, China

**Keywords:** South China Sea, Daya Bay (DYB), water quality, phytoplankton, multivariate analysis, human activities

## Abstract

Data collected from 12 stations in Daya Bay in different seasons in 2002 revealed the relation between water quality and phytoplankton response patterns. The results showed that Daya Bay could be divided into wet and dry seasons by multivariate statistical analysis. Principal component analysis indicated that temperature, chlorophyll *a* and nutrients were important components during the wet season (summer and autumn). The salinity and dissolved oxygen were the main environmental factors in the dry season (winter and spring). According to non-metric multidimensional scaling, there was a shift from the large diatoms in the dry season to the smaller line-chain taxa in the wet season with the condition of a high dissolved inorganic nitrogen and nitrogen to phosphorous concentration ratio. Nutrient changes can thus alter the phytoplankton community composition and biomass, especially near the aquaculture farm areas. There was no evidence of an effect of thermal water from the nearby nuclear power plants on the observed changes in phytoplankton community and biomass in 2002.

## 1. Introduction

Anthropogenic activities have been considered to be the most important factor for the degradation of marine environments, especially in bays and estuarine zones, over the last centuries [1–4]. The main environmental pressures were thought to be pollution from excess nutrient loading, which resulted from agricultural, urban and suburban runoff, wastewater, and air pollution [5–7]. While various indices based on nutrient availability for aquatic primary producers were developed to quantify the water quality [8–10], nutrient levels alone may not be sufficient to indicate the eutrophication or degradation of the environment for some coastal areas, as there are many other factors that determine the ultimate level of the nutrients within an estuary or bay, including tidal exchange, freshwater inflows and water residence time [8,11,12]. Phytoplankton communities are important sentinels of environmental changes, since they integrate the effects of increased nutrient loads, and they can be more sensitive to the combined impacts of stressors than a single stressor [13–16]. In the coastal zone, the water quality and its biological communities are submitted to enormous spatiotemporal variations, caused by natural factors and anthropogenic activities. Identifying the key variables cannot be easily achieved by traditional ecology methods. In contrast, multivariate statistical analysis through clustering and ranking based on species and environment data could successfully identify the key factors from the environmenal variables in the marine environment and other aquatic ecological research [1,17–22].

Daya Bay (DYB) is located in the subtropical ocean, and it is one of the largest and most important gulfs along the southern coast of China. As a subtropical coastal bay, and the dominant ecotypes of phytoplankton in DYB are alongshore warm-water species, highly abundant during spring and summer, and the second dominant ecotypes are marine warm-water species and eurytopic species, found with higher frequency during winter and autumn. *Bacillariophyta* constitute more than 70% of the community, and *Dinophyta* is the second dominant community [23]. The ecological environment of DYB has been significantly impacted by human activities [1,24]. Nutrients changes strongly influence the phytoplankton in this area [24]. Red tide events have occurred more frequently in the waters near Aotou harbor and Dapeng Cove. The phytoplankton structure and its relationships with the environmental factors in DYB have been widely studied [17,24–26]. In the aquaculture farm areas, the conditions of water temperature, salinity, as well as quick recycling of nutrients, play important roles on the high abundance of phytoplankton and frequent blooms in DYB [17,25,27]. Near the nuclear power plants, the concentration of chlorophyll *a* was higher than the concentration around other areas [28], and phytoplankton abundance went up distinctly during the end of autumn and the beginning of winter. The amount of dinoflagellates and warm water species generally increased and net-phytoplankton generally decreased near the nuclear power plants [29]. Previous studies on the phytoplankton were focused only on part of the DYB area, and no detailed studies have been carried out on seasonal variation of environmental factors and patterns of phytoplankton community response in all the areas of the bay. The aim of the present study was to determine the environmental status and the mechanisms of phytoplankton response patterns using multivariate statistical techniques in order to identify and study the relationship(s) between the water quality and phytoplankton community structures, and also to identify the anthropogenic effects of the nuclear power plants and fish farming.

## 2. Materials and Methods

### 2.1. Sampling Design and Analysis of Samples

**Figure 1:**
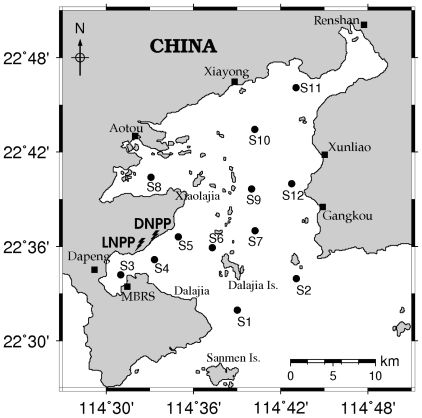
Sampling stations in Daya Bay (Adapted from [17,24]).

Daya Bay is a semi-enclosed bay, located at 114°29′42″–114°49′42″E and 22°31′12″–22°50′00″N (Figure 1). It covers an area of 600 km^2^, with a width of about 15 km and a north–south length of about 30 km. About 60% of the area in the Bay is less than 10 m deep. Dapeng Cove (location of station 3 in this work), in the southwest portion of Daya Bay, is about 4.5 km (N–S) by 5 km (E–W). Located in a subtropical region, the annual mean air temperature in Daya Bay is 22 °C. The coldest months are January and February, having a monthly mean air temperature of 15 °C, and the hottest months are July and August, having a monthly mean air temperature of 28 °C. The minimum sea surface temperature occurs in winter (15 °C), while the maximum occurs in summer and fall (30 °C). No major rivers discharge into Daya Bay, and most of its water originates from the South China Sea. There are only three small rivers (Nanchong River, Longqi River and Pengcheng River) that discharge into Dapeng Cove. The Pearl River is to the west of Daya Bay. Daya Bay Nuclear Power Plant (DNPP) and Lingao Nuclear Power Plant (LNPP) have been operated since 1993 and 2003, respectively [24].

Seasonal surveys were carried out in 2002 at 12 stations (Figure 1) [17,24]. A Quantar_Water Quality Monitoring System (The Hach Company, Loveland, CO, USA) was employed to collect the data for temperature, pH, salinity and depth of water at all stations. Seawater samples for nutrients and chlorophyll *a* were collected and analyzed according to “*The specialties for oceanography survey*” (GB12763-91, China).

### 2.2. Data Analysis

Environmental data was auto-scaled in order to avoid misclassification due to wide differences in data dimensionality. The data was normalized with mean and variance of zero and one, respectively. The eigenvalues and eigenvectors from the covariance matrix of original variables were obtained by principal components analysis (PCA). The eigenvalues of the PCs are the measure of their associated variance, the participation of the original variables in the PCs is given by the loadings, and the individual transformed observations are called scores [1].

For non-metric multivariate analyses of community structure, a similarity matrix was constructed from ln(x + 1) transformed phytoplankton abundance data, using the Bray-Curtis coefficient of similarity and sample interrelations were mapped by non-metric multidimensional scaling (NMDS) [20]. Axes scores of NMDS were accepted as the best descriptors of phytoplankton community structure in two-dimensional space. The correlation of primary symmetric matrix (phytoplankton community) with all subsets of second (environment factors) matrix was calculated in addition to NMDS analysis [20]. The calculation was carried out using MATLAB R2008b (Mathworks Inc., Natick, MA, USA).

## 3. Results

### 3.1. Seasonal and Spatial Variation of Environmental Parameters

**Figure 2:**
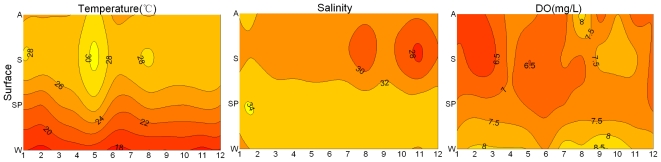

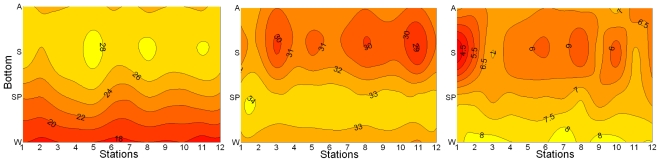
Changes of temperature, salinity and dissolved oxygen (DO) at the surface and bottom water (W: winter, SP: spring, S: summer, A: autumn).

The lowest seasonal average temperature in DYB was recorded in winter (17.9 °C), and the highest occured in summer (27.8 °C; Figure 2-temperature). The surface temperature was 1–2 °C higher in S5 near the nuclear power plants than in the other areas of DYB, due to the discharge of the waste warm water from the nuclear power plants.

The seasonal changes of dissolved oxygen (DO) in DYB were from 6.49 to 8.04 mg/L (Figure 2-DO), and it was higher in winter than in the other seasons. In 2002, the precipitation ranged from 4.0 to 397.5 mm, being lower in winter and spring (data not shown). It is one of the main factors that lead to a seasonal change in salinity (Figure 2-salinity), as there are only three small rivers that discharge into Dapeng Cove.

**Figure 3:**
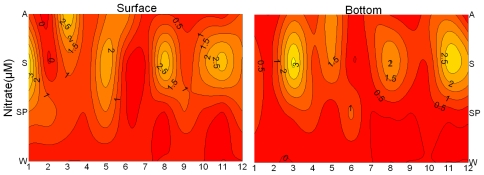

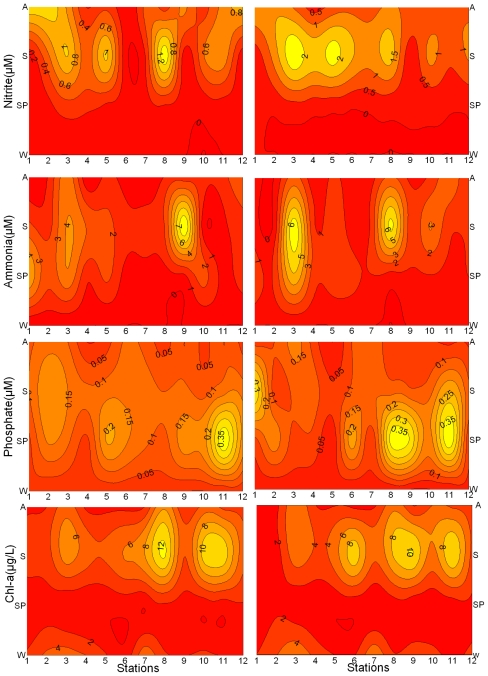
Changes of the nutrient concentration and Chl-*a* at surface and bottom (W: winter, SP: spring, S: summer, A: autumn).

The nutrient concentration distributions and Chl-*a* are shown in Figure 3. The concentrations of nitrate (NO_3_-N) were the highest in summer (Figure 3). In the dry season, NO_3_-N decreased from the mouth to the inner bay. In contrast, NO_3_-N decreased with the distance offshore (except for S 1) during the wet season, as the precipitation and runoff in-puts increased the nutrient flux. During the wet season, the concentrations of NO_3_-N were higher at S3, S8 and S11 (near the aquaculture farming area) and at S5 (near the nuclear power plants) than other stations, which revealed the impacts of human activities in these areas. NH_4_-N and NO_2_-N followed a similar distribution trend. The spatiotemporal distribution of PO_4_-P differed with the dissolved inorganic nitrogen, with high concentrations in spring, although the precipitation was very low in spring. During the wet season, the concentration of PO_4_-P decreased from the mouth to the inner bay (Figure 3). Silicate showed a similar seasonality as dissolved inorganic nitrogen, and the highest value was at S11 (data not shown). The ratios of DIN to PO_4_-P varied in the different seasons, with the lower values in winter (17.89) and spring (28.43), and the higher values in summer (39.32) and in autumn (44.7).

**Figure 4:**
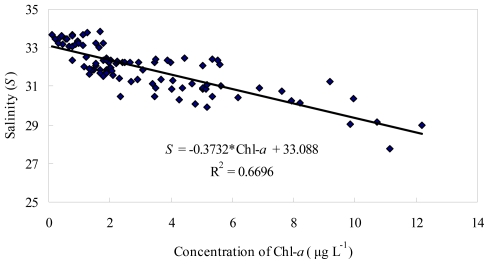
Relationship between Chl-*a* and salinity.

The concentrations of Chl-*a* were the lowest in spring, and the highest in summer (Figure 3). Higher Chl-*a* concentrations were observed in the aquaculture zone (S3, S8, S11) than in other areas of the bay, with maximum at S8 in summer. There was a negative correlation between the Chl-*a* concentration and the concentration of PO_4_-P (r = −0.503, p < 0.01) during the dry season. The phytoplankton biomass was negatively correlated with salinity in 2002 (r = −0.818, p < 0.01) (Figure 4). Temperature, DIN, NO_3_-N, NO_2_-N and silicate were positively correlated with Chl-*a* concentration (p < 0.01).

### 3.2. Principal Component Analysis

#### 3.2.1. The Loadings of Water Quality Parameters on the First Four PCs

The loadings of the four retained PCs are shown in Table 1. On the surface, PC1 (56.2% of the variance) was mainly contributed by T (with a loading of 0.7754), and also indicated that temperature was one of the most important indicators in 2002. PC2 (12.4% of the variance) was mainly contributed by salinity, DO, Chl-*a* and SiO_3_-Si. The loading of salinity was −0.4744, while Chl-*a* gives a positive loading with 0.5779, indicating that there was a negative correlation between salinity and Chl-*a*. PC3 and PC4 were mainly contributed to by BOD_5_, COD and DIN, which revealed the anthropogenic influences. Compared with the loadings of the parameters at the surface, the loadings of TP, NO_3_-N, NH_4_-N and NO_2_-N at the bottom contributed more weight on the first four PCs. PC2 was contributed by TP, PO_4_-P and Chl-*a* at the bottom. Based on the results of the principal component analysis, we conclude that temperature, salinity and DO were the main parameters influencing the environment, and the Chl-*a* was the most important biological variable.

#### 3.2.2. The Effect of Station Score on the First Two PCs

The temporal variation patterns on the surface and bottom exhibited great contrasts between the wet season and the dry season due to the huge (several hundred-fold) precipitation difference. The PCs not only identified the seasonal variation of principal environment components, but also discriminated between natural and anthropogenic effects on changes in the environment factors.

**Figure 5:**
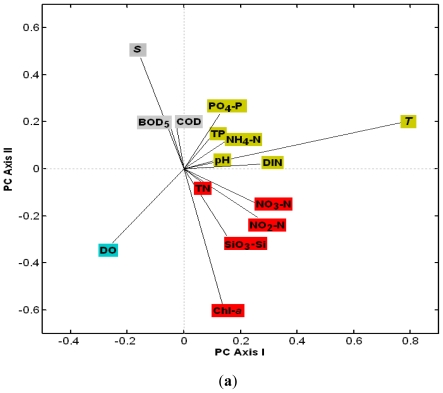

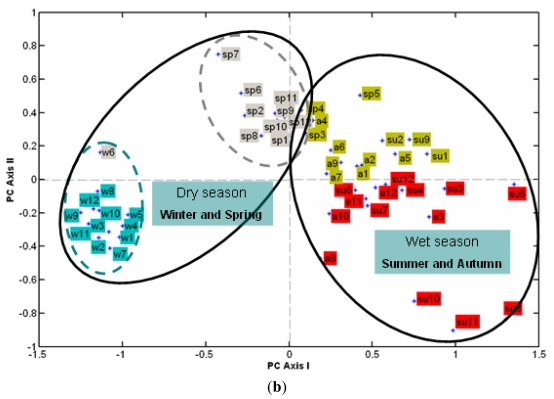
Principal component analysis (PCA) (Axis I and II) made on the loadings of environment variables (**a**) and the scores of the 12 stations in four seasons (**b**) in surface.

**Figure 6:**
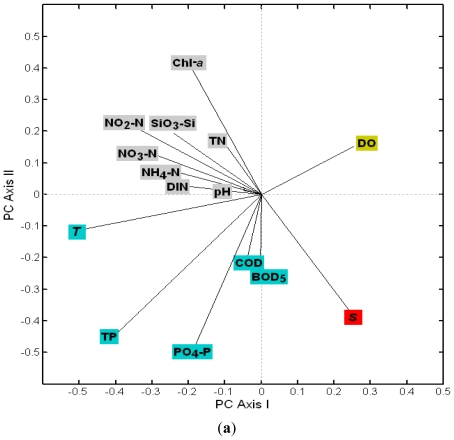

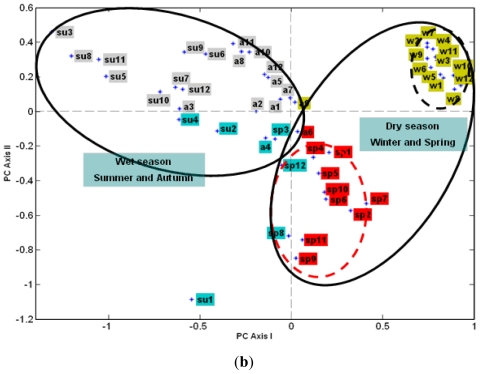
Principal component analysis (PCA) (Axis I and II) made on the loadings of environment variables (**a**) and the scores of the 12 station in four seasons (**b**) in bottom.

The spatial difference of DO, salinity and temperature (except near the nuclear power plants) was not significant in winter. According to the principal component analysis (PCA) results (Figure 5 and Figure 6), the scores of all stations at the surface were relatively uniform in winter, and distributed in the third quadrant of the surface (Figure 5b) and in the first quadrant of the bottom, respectively (Figure 6b). Dynamic mixing and the decrease of temperature in winter led to higher DO. According to Figure 5 and Figure 6, quadrantal distributions of DO variable loading and the stations scores in winter were within the same quadrant, which indicated that DO contributed to PCA more than the other environment factors in winter.

The precipitation in 2002 was lower compared with other years, especially in spring, which resulted in a higher average salinity. According to the PCA result, the scores of all stations in spring were scattered in the same coordinate space with the salinity loading (Figure 5 and Figure 6). The concentration of Chl-*a* had a significantly negative correlation with salinity (r = −0.818, p < 0.01), coinciding with the negative distribution along the PC Axis II between salinity and Chl-*a* (Figure 5a and Figure 6a).

During the wet season, the temperature was higher than during the dry season. Moreover, high precipitation and human activities lead to high nutrient input into DYB, inducing high Chl-*a*. The temperature, nutrients and phytoplankton biomass were the most important environmental factors in the wet season. They contributed more weight in the PCA results (Figure 5 and Figure 6). Both the scores of wet season and the loadings of nutrients, temperature and Chl-*a* were correlated with the first axis positively on the surface (Figure 5) and negatively at the bottom (Figure 6), respectively. The PCA could identify the impact of anthropogenic activity on the phytoplankton biomass. For instance, both Chl-*a* variable and the scores of the su8, su11 su10, a8, and a3 were most negatively correlated with the axis II in the wet season on the surface (Figure 5a, b). It revealed that the above stations were influenced by the aquaculture farm and freshwater runoff, characterized by a higher level of Chl-*a* than the other stations around the bay. According to Figure 6b, S1, S2 and S4 were exceptional stations in summer. S1 is located near the west side of the bay mouth and had very low DO and high COD, BOD_5_, TP and PO_4_-P. S2 is located on the east side of the mouth and featured the strongest stratification with low temperature and high salinity in the bottom layer. Consequently the scores for S1 and S2 at the bottom were different from those of other stations in summer (Figure 6b). The bottom water at S4 was near the intake of the DNPP cooling system. The entrapment of organisms into the pumps and pipes of the cooling system of DNPP could be lethal, as proved by lower Chl-*a* than the other nearshore areas, therefore the score of S4 in summer and autumn was negatively selected by Axis II (Figure 6b). In summer, the score of S5 in summer was the most positively related with first axis (Figure 5b), corresponding to the temperature in Figure 5a, as the most important factor contributed to the first PC axis. It indicated that the temperature was the key factor influencing the environment at S5.

### 3.3. Phytoplankton Community Structure

**Figure 7:**
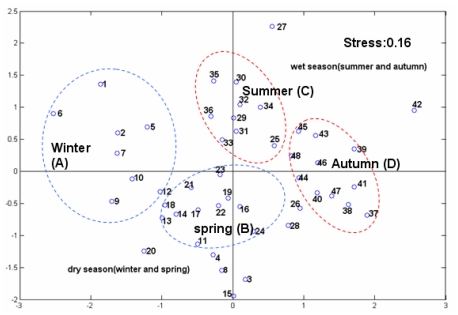
NMDS of phytoplankton community structure (numbers from 1 to 12 represent winter stations 1–12; numbers from 13 to 24 represents spring stations 1 to 12; numbers from 25 to 36 represent summer stations from 1 to 12; numbers from 37 to 48 represent autumn stations from 1 to 12).

The Bray-Curtis coefficients of similarity and sample interrelations were mapped by non-metric multidimensional scaling analysis. The seasonal stations are separated in the plane. The phytoplankton community showed wet and dry season contrasts (stress, 0.16) along the first axis of NMDS ordination (Figure 7). The first axis of NMDS was significantly correlated with Chl-*a* (p < 0.05), temperature (p < 0.05) and DO (p < 0.01). The second axis was correlated with salinity (p < 0.05), DIN (p < 0.05), PO_4_-P (p < 0.05), SiO_3_-Si (p < 0.05), and DIN:PO_4_-P (p < 0.01).

During winter, the low temperature and high salinity of DYB due to intrusion of the sea water enhanced the growth of eurytopic species and seawater species in DYB (Group A). Group A was mainly composed of *Chaetoceros eibenii*, *Thalas–siothrix fraenfeldii* and *Bacillaria paradoxa Gmelin. Chaetoceros eibenii* was the most abundant species and its abundance was significantly correlated with DO (r = 0.840, p < 0.01), PO_4_-P (r = −0.683, p < 0.05) and the DIN:PO_4_-P ratio (r = 0.588, p < 0.05). In spring, the dominant species was ascribed to Group B. *Chaetoceros lorenzianus* was the most dominant, and it’s abundance was significantly correlated with NO_2_-N (r = 0.811, p < 0.01), NH_4_-N (r = 0.982, p < 0.01) and PO_4_-P (r = 0.733, p < 0.01). In Dapeng and Aotou Cove, the phytoplankton communities of S3, S4 and S8 (number 3, 4, 8, 15 and 20 in Figure 7) were different from the other parts of the bay during the dry season.

The wet season in DYB was characterized by high temperature, low salinity and high content of nutrients, such as DIN, and these three factors determined the dominant species exhibiting nitrophilicity characteristics and tolerance to high temperature and lower salinity during the wet season (Group C). The main phytoplankton ecotypes were alongshore species, such as *Pseudo-nitzschia delicatissima, Skeletonema costatum* and *Thalassionema nitzschioides*. The density of *Pseudo-nitzschia delicatissima*, the most dominant species in summer, showed a significant correlation with NO_3_-N (r = 0.593, p < 0.05), NO_2_-N (r = 0.830, p < 0.01), NH_4_-N (r = 0.636, p < 0.05) and DIN:PO_4_-P (r = 0.810, p < 0.01). *Skeletonema costatum* and *Thalassionema nitzschioides* were the dominant species in autumn, and were ascribed to Group D. The density of *Skeletonema costatum* was significantly correlated with NO_3_-N (r = 0.710, p < 0.01) and NH_4_-N (r = 0.707, p < 0.01.) during autumn.

## 4. Discussion

PCA and NMDS could distinguish the natural and anthropogenic factors that influenced the phytoplankton community. The hydro-physical environment factors (temperature, salinity) controlled the phytoplankton ecotype, and the nutrients were related with the biomass and the composition community, especially in the wet season.

Previous studies demonstrated that nutrients were the main factors determining the concentrations of chlorophyll *a* and phytoplankton community in coastal waters [30–32]. In case of Daya Bay, the highest chlorophyll *a* concentrations were recorded at station S8, and the next at S11 and S3. Those stations are located in the fish and shellfish cage culture areas. Nutrient levels were higher at these stations than elsewhere, indicating that the release of amount of nutrients from fish farming stimulated the growth of phytoplankton. These results are also supported by the affinity between Chl-*a* and the nutrients in the loadings distribution (Figures 5–6). There is a large thermal plume extending 8–10 km south along the coast from the nuclear power plants [28]. According to the score distributions and the loadings of environment factors, the score of S5 was most relevant with the loading of temperature. It indicated that the direct effect of the nuclear power plants on the environmental changes was due to the increase in the temperature [24]. The biomass and the density of phytoplankton were lower at S4 and S5 than the elsewhere near the shore area (S3, S8 and S11) in this study. This result was also supported by Wang *et al.* [24]. This revealed that there were more contributions to the phytoplankton biomass in Daya Bay by aquaculture than by the effect of the nuclear power plants. Although the community structure of S4 in summer (number 28 in Figure 7) was different from the others based on the NMDS results, low species diversity and evenness were recorded at S4, but also at S2 and S3 in summer [23], indicating that the differences between S4 and the other stations may not only be the result of the effect of the nuclear power plants. Previous studies showed that the Chl-*a* biomass and the community structure were significantly impacted by the nuclear power plants [28,29]. In contrast, the influence of the nuclear power plants on the phytoplankton biomass and community was not obvious in 2002. Tang *et al.* reported that the concentration of Chl-*a* at S4 was the highest in the entire bay in 1997 and 1998 [28], and a strong El Niño event also occurred in this period. It was tentatively put forward that there may be an impact for serious El Niño events on the phytoplankton in 1997 and 1998 in Daya Bay [17,24,33]. Liu *et al.* reported that the amount of dinoflagellates and warm water species generally increased and net-phytoplankton generally decreased near the nuclear power plants from 1991 to 2001 [29], but these phenomena reported by Liu *et al.* [29], also occurred in the entire bay from 1982 to 2002 [24]. This may be resulted by a shift from low DIN: PO_4_-P to high ratios of DIN: PO_4_-P and Si:P in recent years. It should be noted that the bottom water at S4 was near the intake of the cooling system of the nuclear power plants, and entrapment of organisms in the pumps and pipes of the cooling system of the nuclear power plants could be lethal and affect the measured populations. Long time scale monitoring is needed to assess the potential effects of the nuclear power plants on the environmental and organism changes.

The seasonal succession of dominant species revealed a shift from large species during the winter and spring to a progressive dominance of the smaller chain-forming taxa such as *Pseudo-nitzschia delicatissima, Skeletonema costatum* (<20 μm), and the shift was depended on the ecological habit, temperature, salinity and nutrient variation. In winter and spring (the dry season), the dominant species were marine warm-water species and eurytopic species. In contrast, the phytoplankton community structures at S3 S4, S8 and S11 were different from the other stations by NMDS, whereby the community composition was mainly alongshore warm-water species, and the no significant dominant species were identified [23]. In spring, the concentrations of PO_4_-P and NH_4_-N were higher than those in winter. These factors were important environmental components according to PCA in spring because the loading of PO_4_-P and NH_4_-N and the station scores in spring were both correlated with the PC Axis II, and they were the key candidates in the environmental factors for the growth of the spring dominant species according to the NMDS results. In the wet season, the values of DIN:PO_4_-P increased to about 40, contrasting with the low levels (17.89 in winter and 28.43 in spring) in the dry season, and also indicating that PO_4_-P became the limiting nutrient [24]. Under this limited PO_4_-P condition, the dominant species were the smaller chain-forming taxa *Pseudo-nitzschia delicatissima, Skeletonema costatum* and *Thalassionema nitzschioides*, which also showed that nitrophilicity and tolerance to high temperature and lower salinity during the wet season. It is known that under continuous nutrient limitation the smaller phytoplankton species appear to be better competitors for nutrients and light than larger ones due to their surface/volume ratio [34].

The composition of dominant species and the sequence of dominance have changed in Daya Bay. The changes of nutrient concentration may be the key factors accelerating the progress of the succession of dominant species in Daya Bay. The primary dominant species during summer in 1983 and 1991–1995 were the *Chaetoceros* genus [26,29]. During the summer of 2002, the dominant species were *Pseudo-nitzschia delicatissima, Skeletonema costatum* and *Thalassionema nitzschioides.* The genus *Chaetoceros* was dominant during spring. Under the condition of high concentration of DIN and high ratio of DIN:PO_4_-P, *Pseudo-nitzschia delicatissima* and *Skeletonema costatum* exhibited stronger competition for nutrient uptake than *Chaetoceros* [35]. The concentrations of nitrate and DIN: PO_4_-P were lower in spring than in summer, which may explain why the numerical dominance of *Chaetoceros* deceased from spring to summer. The magnitude of changes in the ecological environment in Daya Bay were obvious during the past two decades, especially with respect to the total nitrogen, phosphorus, and the ratio of DIN:PO_4_-P [17]. The requirement of nutrients for deferent species varied according to individual physical and ecological features, thereby demonstrating that the nutrients played an important role in determining the variations in the phytoplankton density and the species succession in Daya Bay.

## 5. Conclusions

In summary, Daya Bay could be divided into wet and dry seasons by the multivariate statistical analysis based on environmental variables. According to the PCA results the important components included temperature, chlorophyll *a* and the nutrients during the wet season (summer and autumn), and salinity and dissolved oxygen in the dry season (winter and spring). According to non-metric multidimensional scaling, the phytoplankton community was different from the wet season to the dry season. A succession from the large diatoms in the dry season to the smaller line-chain taxa in the wet season occurred under the conditions of the high DIN and DIN:PO_4_-P concentration. Nutrients varied spatially and temporally, and altered the phytoplankton community composition and biomass. In the areas influenced by anthropogenic activity (S3, S8 and S11), the higher nutrients led to higher content of phytoplankton biomass. There was no evidence for an effect of thermal water from the nuclear power plants on the changes in the phytoplankton community and biomass in 2002. The larger scale petrochemical plants near DYB were only developed in recent years and their influence on the environmental changes should be addressed in future research.

## Figures and Tables

**Table 1:** Loadings of water quality parameters on the first four PCs.

Parameter	Surface				Bottom			

	PC1	PC2	PC3	PC4	PC1	PC2	PC3	PC4
T	0.7754	−0.1987	0.0125	0.3023	0.502	−0.1127	0.2514	−0.0756
pH	0.102	−0.0329	0.1233	0.1385	0.0497	0.0132	0.0119	−0.0163
S	−0.1539	−0.4744	−0.1255	−0.1125	−0.2428	−0.3668	0.0177	−0.3448
DO	−0.2535	0.316	0.1568	−0.1276	−0.2547	0.1529	−0.2819	−0.0906
BOD_5_	−0.0469	−0.1745	0.6619	0.0597	0.0028	−0.2294	0.0098	0.4245
COD	−0.0257	−0.1801	0.6116	0.1161	0.0409	−0.2141	0.1092	0.425
Chl-*a*	0.1359	0.5779	0.1367	0.031	0.1927	0.4013	−0.3423	0.4434
TN	0.0608	0.0569	0.2448	−0.2493	0.1071	0.1702	−0.3496	−0.2304
NO_3_-N	0.2517	0.1459	0.0284	−0.4487	0.3024	0.1301	−0.2491	−0.0113
NO_2_-N	0.2622	0.2086	−0.1022	0.0624	0.3559	0.2176	0.2283	−0.2461
NH_4_-N	0.1475	−0.1194	0.0692	−0.4392	0.2372	0.0732	−0.2411	−0.2637
TP	0.0933	−0.1334	0.0551	−0.1064	0.4013	−0.4433	−0.3708	−0.1655
PO_4_-P	0.1275	−0.2349	−0.1349	0.0523	0.1842	−0.4857	−0.1892	0.2221
SiO_3_-Si	0.1525	0.2863	0.1133	0.2308	0.2428	0.1948	0.1607	0.2049
DIN	0.2709	−0.0199	0.0579	−0.5611	0.2077	0.0269	0.4838	−0.087
Variance (%)	56.19	12.39	9.26	7.81	36.21	14.09	6.87	6.09
Cumulative (%)	56.20	68.58	77.84	85.65	36.21	50.30	57.17	63.26
